# Environmental, Economic, and Social Aspects of Human Urine Valorization through Microbial Fuel Cells from the Circular Economy Perspective

**DOI:** 10.3390/mi13122239

**Published:** 2022-12-16

**Authors:** Mariana Martínez-Castrejón, Jazmin A. López-Díaz, Omar Solorza-Feria, Oscar Talavera-Mendoza, América L. Rodríguez-Herrera, Osbelia Alcaraz-Morales, Giovanni Hernández-Flores

**Affiliations:** 1 Centro de Ciencias de Desarrollo Regional, Universidad Autónoma de Guerrero, Privada de Laurel No. 13, Col. El Roble, Acapulco C.P. 39640, Guerrero, Mexico; 2 Escuela Superior de Ciencias de la Tierra, Universidad Autónoma de Guerrero, Ex hacienda San Juan Bautista s/n, Taxco el Viejo C.P. 40323, Guerrero, Mexico; 3 Centro de Investigación y de Estudios Avanzados del Instituto Politécnico Nacional, Department of Chemistry, Av. Instituto Politécnico Nacional 2508, Col. San Pedro Zacatenco, Delegación C.P. 07360, Gustavo A. Madero, Mexico; 4 Facultad de Arquitectura y Urbanismo, Universidad Autónoma de Guerrero, Av. Juárez No. 38 Interior. C.U. Zona Norte, Chilpancingo C.P. 39000, Guerrero, Mexico; 5 CONACYT-Escuela Superior de Ciencias de la Tierra, Universidad Autónoma de Guerrero, Ex Hacienda San Juan Bautista s/n, Taxco el Viejo C.P. 40323, Guerrero, Mexico

**Keywords:** bioelectrochemical systems, circular economy, electrochemical systems, human urine

## Abstract

Population growth increases the challenge of meeting basic human needs, such as water, a limited resource. Consumption habits and water pollution have compromised natural resources to unsustainable levels. Sustainable effluent treatment practices, such as decentralized systems focused on energy, nutrients, and water recovery, have attracted the attention of the scientific community. Human urine (HU) is a physiological liquid waste whose main component is water (~95%). HU has a significant amount of nutrients, such as N, P, K, and organic matter, which are usually lacking in fecal coliforms. Therefore, the possibility exists of recovering nutrients and energy from HU using sustainable and non-sustainable technologies. Treating HU in bioelectrochemical systems (BES) is a novel alternative to obtaining byproducts from this effluent more sustainably than in electrochemical systems. Microbial fuel cells (MFCs) are an interesting example, contributing to HU revalorization from unwanted waste into a valuable resource of nutrients, energy, and water. Even when urine-operated MFCs have not generated attractive potential outputs or produced considerable amounts of bioelectricity, this review emphasizes HU advantages as nutrients or water sources. The aim of this review was to analyze the current development of BES for HU treatment based on the water circular economy, discussing challenges and perspectives researchers might encounter.

## 1. Introduction

Water is a key and finite natural resource for life. Quality freshwater is essential for the proper functioning of human metabolism. Furthermore, water is essential for countless production processes, i.e., water is an indispensable resource for the existence of humanity and its socioeconomic development [1]. Unfortunately, water quality due to excessive and irrational consumption has deteriorated alarmingly [2,3]. Water limitations have caused society to seek different strategies to stock up on this vital fluid. Specific treatments focused on removing contaminants have been developed to recover the quality of wastewater (WW) and reuse it [4]. Anthropogenic activities produce a variety of WW with complex compositions that, fortunately, can be treated to recover quality.

Human urine (HU) is an abundant physiological effluent. Considering a world population of eight billion, it is calculated that ~20,000,000 m^3^ of HU/day are produced [5] with the potential to be an unconventional source of water, energy, and nutrients when treated by bioelectrochemical systems (BES) [6,7,8,9].

Being a metabolic waste generated from freshwater in its different physiological processes, HU is generally free of enteropathogenic bacteria such as Escherichia coli [10,11]. As a reflection on people’s health and habits, HU composition varies but broadly can be divided into water and dissolved solids (DS), at ca. 95 and 5%, respectively. The highest DS concentration is due to urea, inorganic salts, organic compounds, and organic ammonium salts [12]. Due to the high content of N and P, HU composition poses a challenge for wastewater treatment plants (WWTP) [13]. Nevertheless, N and P are two essential elements for plant nutrition that can be recovered by BES or directly aspersed [9]. Additionally, using BES, it is possible to use the organic compounds present in HU as fuel within these devices and generate electrical energy [9,13]. Once N, P, and K have been removed from HU and organic matter has been converted into an energy source, HU can be subjected to additional treatment to recover water. This biotechnological approach transforms a problem effluent into a potential solution, increasing water use efficiency by adding value to HU components and enabling as many reuses as quality permits. These actions are within the philosophy of a new concept: “water circular economy” [4]. In this review, we adopt this novel approach to reinforce BES sustainability by highlighting residue revalorization and water recovery to address HU complete reuse. Due to its abundance, physicochemical, and microbiological characteristics, HU can be considered a solution as a source of water, energy, and macronutrients obtained through the use of BES.

## 2. Materials & Methods

An online search in Academic Google was carried out by looking for papers including the concepts: “urine treatment” and “water recovery”, obtaining 279 results ordered by relevance; and “human urine”, “microbial fuel cell”, and “recovery”, retrieving 882 results. From these searches, papers focused on real HU treatment were selected. A second distinction among papers was made to focus on water recovery technology results. The outcome brought seven review articles focused on HU treatment, but only one of them was focused on water reclamation. None of the articles reviewed for this work were found to be based on the water circular economy or to address economic, environmental, or social issues, i.e., the sustainable perspective.

## 3. Results

### 3.1. Circular Economy

**Figure 1:**
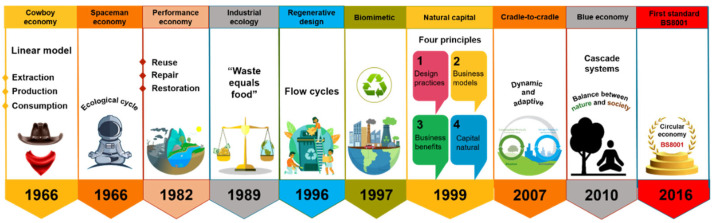
Circular economy concept development over time.

**Figure 2:**
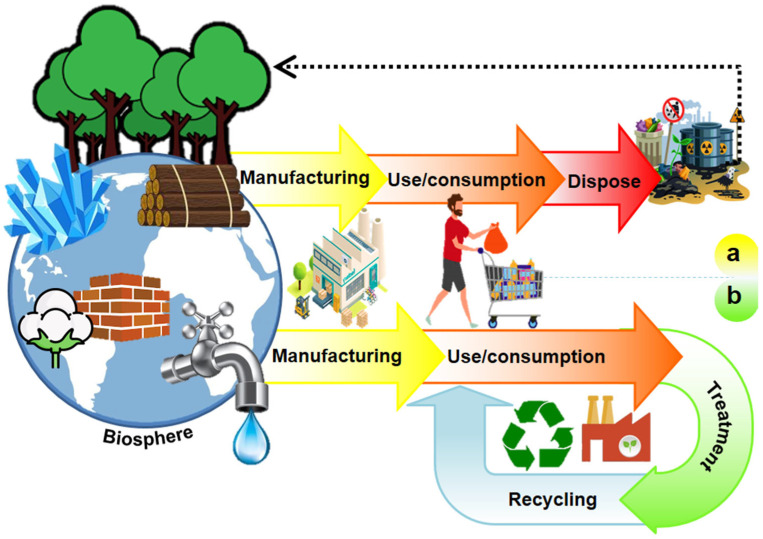
Economic models: (**a**) linear economy and (**b**) circular economy.

In the past decade, the concept of circular economy (CE) has attracted a vast audience, i.e., stakeholders, policymakers, citizens, media, and academics. It is emerging as the appropriate paradigm to achieve the transition to sustainable development-compatible models (Figure 1). Due to the negative impacts that the unsustainable take-make-dispose system has had on the environment (Figure 2a), the CE is proposed as a philosophy to reduce the environmental pressures the linear economy has driven and as the appropriate paradigm to achieve the transition to sustainable development-compatible models [14,15]. The main interest of this approach is to make the best use of available material and cultural resources. The waste generated in producing a good becomes raw material to create a new product and continue the productive flow (Figure 2b). Reusing waste depends on the social acceptance and cultural revalorization that can be achieved. Because it is a holistic approach to resource management oriented toward sustainability, the systemic approach is pointed out as the ideal theoretical perspective for the valuation of this paradigm [14,16].

#### Water Circular Economy

**Figure 3:**
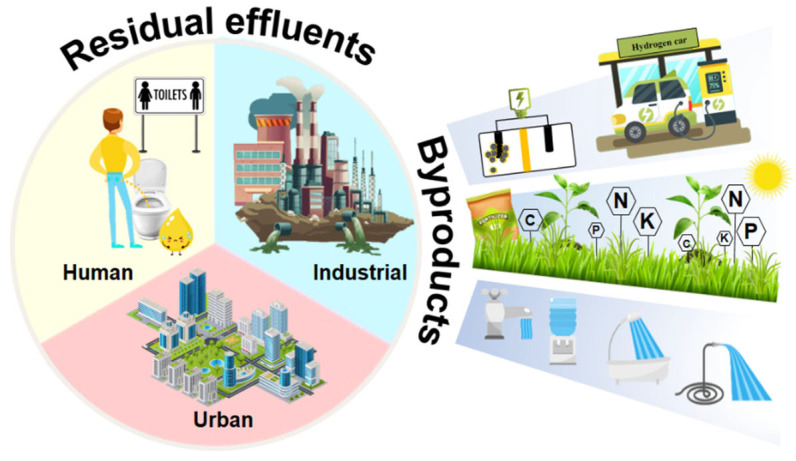
Residual effluents and their byproducts.

The imperative need for water, global scarcity, unavailability, and the high cost of its purification and treatment have driven the restoration and reuse of a variety of anthropogenic WW in search of socially valuable byproducts (Figure 3).

**Figure 4:**
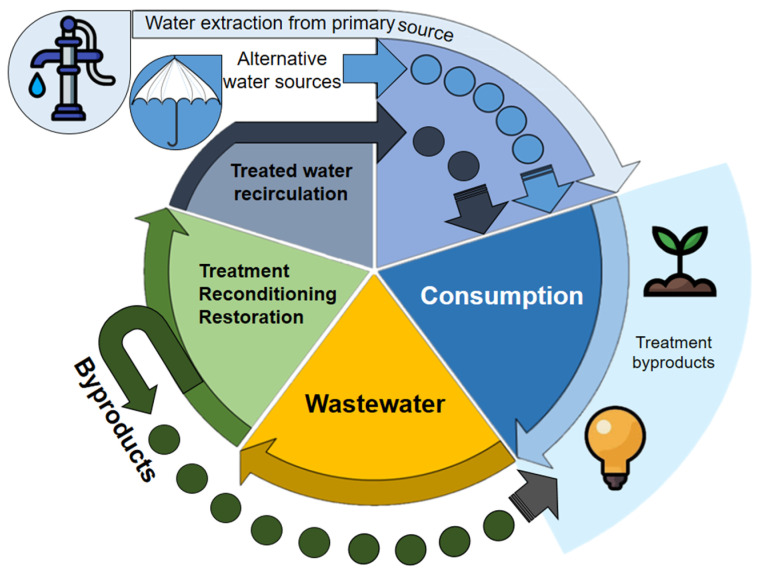
The circular economy of water.

CE discourages the extraction of virgin resources, prioritizing waste treatment. The complete cycle of WW management is a critical component for water CE, from its generation, collection (on-site sanitation systems and sewers), and treatment for its disposal and reuse. The water CE aims to close water, nutrient, and energy flows to extend their useful lives through use, reuse, and recovery processes [17,18]. This transition encourages efficient water use, combined with strong incentives for innovation, which can improve the ability of the economy to handle the demands of a growing imbalance between water supply and social demand [19]. Water reuse is an alternative supply to satisfy human needs globally [4]. From a CE perspective, reusing water is a beneficial option for both humans and the biosphere. Addressing the increasing demand for resources will require a combination of approaches, including water conservation, recycling, WW treatment, and non-traditional sources such as rainwater and treated HU (Figure 4) [20].

### 3.2. Human Urine

Urine is a waste product of the human body and mammals [21]. HU is a liquid waste produced by the kidneys [10,11]. It is composed of water, urea, electrolytes, organic acids, toxins, compounds resulting from the breakdown of blood, heavy metals, and metabolites in general, which a person ingests through food, water, or exposure to the environment [10,22]. This physiological effluent, a result of the metabolism, has been used to evaluate the health condition of people since the ancient inhabitants of Egypt, Babylon, and India [21]. Furthermore, due to its composition, HU has characteristics that favor its revalorization from the water CE perspective.

The physicochemical characteristics of urine are due to the composition of this physiological effluent. An average fresh HU is transparent and has a pH ranging from 5.5–7.0 with high electrical conductivity (160–270 mS/cm) due to a high content of salts [12]. However, the decomposition of urea into ammonia by the effect of bacterial growth at room temperature modifies the pH from acid to alkaline [21].

Two major components can be observed at HU: water and DS. Water is the component with the highest concentration. The water content is in an average range of 91–95%, and the rest represents the content of DS. The highest concentration of DS is due to the presence of N (14–18%), C (13%), P (3.7%), and K (3.7%). Nitrogen, as urea, is the main component of DS, and its concentration reaches 8.12 g/L. Urea is the predominant solute, representing ca. 50% of the total dissolved organic solids, and represents an important nitrogen source. It is possible to obtain between 60–90% of the N, P, and K in solution from urine, which the plants require for their correct development [23].

#### 3.2.1. A Highly Valuable Waste. Human Urine Byproducts

From these characteristics, the perception of urine can be transformed. Its composition can provide the equivalent of 37% N, 20% P, and 15% K of commercial fertilizers used in agriculture [24]. On the other hand, due to the high content of chemical oxygen demand (COD), up to 17.50 g/L, the chemical energy stored in oxidizable compounds can be converted to bioelectricity by BES [7,9]. Finally, through additional processes, once the content of N, P, K, and *COD* has been removed, the remaining water can be subjected to further treatments for recovery or consumed in activities where high quality is not needed, e.g., garden irrigation.

##### Nutrients

The need to feed a rapidly growing society increases annually by ca. 1.5% of the fertilizer demand. Depending on the demographic explosion, the fertilizer demand is expected to increase over the years [25].

HU contains N, P, K, Mg, Na, S, and Ca in ionic form, and to a lesser extent, minor elements such as Cu, Zn, Mn, Bo, and Fe. HU is a potential source of N, P, and K, some of the main macronutrients needed by plants for their proper growth [24,26,27,28,29]. Its availability to plants is comparable to that of chemical fertilizers [23,30].

Recent research proposes the recovery of these components and water from the constantly increasing world volume of municipal wastewater treatment plants (MWWTPs) [31].

The current dependence of the agricultural sector on expensive industrialized technologies constrains water and nutrient recycling, worsens environmental degradation by favoring pollution, and limits economic development [27,32]. HU has been proposed as a substitute for conventional artificial fertilizers or inorganic nutrients [27,33]. It has been used in different edible crops such as tomato, cereals, corn, amaranth, banana, potato, and spinach, among others [26,27,30,34,35,36]. Nutrient recovery from HU reduces the need to produce, acquire, and import chemical fertilizers and prevents high-nutrient-loaded MWWTPs discharges from coming into contact with natural bodies and streams, avoiding eutrophication issues [27,35,36]. Phosphorus recovery through the precipitation of struvite is one of the most developed issues in this aspect of waste recovery [37,38]. Typically, adults excrete between 1500 and 1600 mL of HU in a day. However, it is normal for an adult to generate between 600 and 2000 mL in a 24 h period. Human beings can excrete ~500 L (0.5 m^3^) of urine annually. It is estimated that from this volume, a person can produce between 2.5–4.3 kg of N, ~1 kg of P, and ~1 kg of K. These values are higher than those registered for fecal matter (~0.7 kg N, ~0.5 kg P, and ~0.2 kg K). These macronutrients represent 60–90% of the intake of N, P, and K that plants require [23,34,35,39].

In addition to the recovery of macronutrients, due to the high percentage of water reported in its composition, the recovery and purification of water from urine are fields developed parallelly [40].

##### Water from Human Urine

The main component of HU is water (ca. 95%). Considering a person produces a volume of 2 L of HU/day, the amount of water that can be recovered from that volume is 1.9 L daily/person. Average per capita HU production per day is estimated to be 1.5 L, with estimated annual production ranging from 300 to 550 L [41]. Considering a world population of 8 billion, it is calculated that 20,000 million L (20,000,000 m^3^) of HU/day, i.e., ~19,000 million L (19,000,000 m^3^) of water, could be recovered daily [5].

Larsen et al. [42] have estimated an average of 184 L (0.184 m^3^) of WW produced per person/day. From this parameter, it is inferred that the world population (8 billion) generates a total of 1,472,000 million L (1,472,000,000 m^3^ or 1,472 hm^3^) of WW/day. Considering the amount of urine produced per capita/day, HU accounts for 1.3% of the WW generated in a 24-h period.

From the water CE perspective, the most efficient approach for the recovery of by-products, particularly nutrients, from waste effluents is integrating systems with separation at the source and decentralized treatments [42,43,44].

The treatment decentralization distinguishes two variants: (i) the totality of the generated WW treatment and (ii) the separated treatment of different residual effluents according to their characteristics, levels, and type of pollution, as well as their potential to be re-used or reincorporated into hydraulic systems, i.e., from a circular perspective [45].

**Figure 5:**
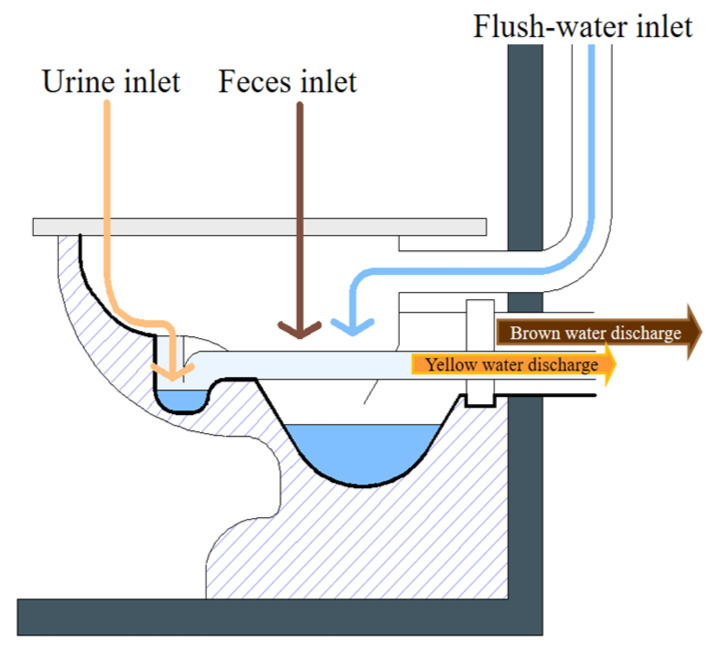
Simple divergent toilet model.

In the domestic environment, separating the WW generated by the conventional toilet (black water) from the soapy WW (gray water) makes the recovery of 80–95% of the nutrients present in human excreta possible. The application of urine separating or diverting toilets allows for the acquisition of two additional types of WW: urine with or without entrainment water (yellow water) and feces with water (brown water) (Figure 5) [45].

Even when the original objective for separating urine from the source was its direct use in garden irrigation or to be stored and then collected to be used as a liquid or processed fertilizer, recent advances have enabled a more sustainable approach that addresses social, economic, and environmental issues such as water savings. To avoid excessive water use, low-flush toilets have been designed to prevent excellent quality water use and reduce the WW quantity received by MWWTP. The separate collection of urine reflects both the saving the liquid and the higher concentration of nutrients [45]. Separating urine from municipal wastewater (MWW) reduces its contamination load by 10, 75, and 50% of COD, N, and P, respectively [42]. Furthermore, this makes it possible to treat HU by itself and increase the efficiency of N and P recovery or oxidation of the COD by decreasing the complexity of the effluent [46,47,48].

Despite the economic and environmental advantages of dry separator toilets, users still need to fully accept them, particularly in urban environments [49]. They tend to remain untidy and have a poor appearance because they lack hydraulic discharge [42]. Separating urine from the source requires special facilities that prevent excreta from combining, representing an additional infrastructure expense compared to conventional systems (Table A1). However, the urine separation allows this effluent to be considered as one of the unconventional sources within the CE.

### 3.3. Technologies for Recovering Water from Human Urine

According to Patel et al. [25], reclaimed water from HU can be used directly for secondary uses such as flushing toilets or even washing, i.e., uses with dermal contact. The authors conclude that the recovered water could be drunk after additional treatments.

Applied membrane technologies with a focus on nutrient recovery remove water from the urine, strip it of contaminants, pharmaceuticals, heavy metals, and nutrients, and obtain water as a byproduct (Table 1). Membrane technologies stand on the application of force through a semi-permeable membrane. Reverse osmosis (RO), nanofiltration (NF), membrane distillation (MD), and forward osmosis (FO) are some examples reviewed in this work [25].

RO is a water purification process using special membranes. In this process, the pressure applied to the target effluent is higher than the osmotic pressure, which favors the retention of salts in the membrane and allows the passage of water and a shallow concentration of salts. Reverse osmosis is used in treating human urine to reduce the volume and concentration of nutrients. In this process, the membrane plays a significant role. One of its main limitations is its deterioration due to the accumulation of trapped particles, which reduces the flow through it and increases the cost of the systems. When these systems treat human urine, obstructions can be generated in the membrane, limiting its useful life and maintaining a constant flow. For this reason, it is necessary to pre-treat the urine before implementing reverse osmosis. Chemical dosing is essential to avoid membrane fouling. In addition, this treatment involves a high energy cost, which translates into operating costs for maintenance [25].

NF has been used in urine to remove micropollutants such as ibuprofen, diclofenac, carbamazepine, ethinylestradiol, and propanol [57]. The membranes used for NF can screen molecules in the 1–10 nm range because their pore size ranges between 1–5 nm [58].

MD utilizes a hydrophobic membrane, which allows only vapor molecules to pass through. This technique uses the pressure gradient principle created by a temperature differential [59]. Due to the characteristics of the process, the temperature affects the transfer of ammonia directly, i.e., the higher the temperature, the greater the transfer of this compound through the membrane. On the other hand, modifications in the properties of the membrane also affect its performance. Tun et al. [51] demonstrated that acidification of HU is paramount to obtaining low ammonia transfer.

The FO process is a low-cost technology for urine treatment and simultaneous recovery of water and nutrients. This process reduces volume and favors the concentration of the nutrients using draw agents (DA), generally water with low chemical potential [25]. The FO process works due to the difference in chemical potential between two solutions (chemical potential gradient). In this system, the water will go from a higher potential gradient to a lower one through an osmotic membrane. These membranes can remove emerging pollutants (EP) such as hormones without consuming energy [60]. FO employs brine as DA to reject N, K, and P up to 50–80%, >95%, and >90%, respectively [61]. FO would be an environmentally friendly process and cost less than other membrane technologies if it were not for the regeneration of DA, which increases the operational energy consumption [25]. Thus, energy consumption depends on the methods used for DA regeneration.

On the other hand, the most commonly used post-treatment of bio-based DAs to recover drinking water has been MD [52,53,62,63]. Another parameter of importance in the treatment of FO is the reverse salt flux. This value reduces the concentration of DA and contributes contaminants to the feed, negatively affecting dewatering efficiency [25].

Urine shows a low water flux of 4.5 LMH due to its high osmolarity [53]. Furthermore, it presents a low chemical potential gradient across the membrane due to the ions and solutes it contains [25]. Urine can be used directly as DA in FO systems due to its chemical potential characteristics and because it can generate osmotic pressure [62].

On the other hand, evaporation and lyophilization are alternative methods to recover water from HU (Table 1). Water removal from urine by evaporation is the simplest method applied. This reduction helps mitigate complications due to urine transportation for its treatment [64]. The method consists of heat application to achieve evaporation (130 °C). However, the increase in temperature represents a limitation for the N recovery due to the volatilization of ammonia. This effect can be minimized by stripping H_2_SO_4_ in ammonium sulfate form or by urine stabilization [65]. The byproduct derived from this treatment is rich in K and P and can be directly used in crop fields [66,67].

Finally, lyophilization/freeze-drying is one method based on crystallization. During the urine freeze-drying process, the formation of the crystals is promoted by increasing the concentration of ions in the solution. The resulting solidified water is free of salts and ions. In the second stage of the process, the ice gets solubilized by applying heat. The difference in water vapor pressure between salt and pure water and the freezing point depression result in the separation of ice and salt solution [56]. The lyophilization process removes odors from the urine and mitigates ammonia losses [68]. Table 2 presents favorable characteristics and areas of opportunity for developing the described technologies.

The HU byproducts recovery techniques have external requirements, i.e., energy consumption, chemical requirements, and/or control systems, to ensure operation (Table 2 and Table A2).

### 3.4. Human Urine as Fuel in Microbial Fuel Cells

Among the technologies implemented for urine treatment, electrochemical and bioelectrochemical treatment stand out, with microbial fuel cells (MFCs) being the most scientifically developed. These devices allow for duality in the treatment by producing bioelectricity, i.e., clean energy, while remediating residual discharges simultaneously [7,69,70].

#### 3.4.1. Human Urine Advantages

Source-separated HU is the optimal substrate for MFCs. It has unique physicochemical characteristics, making it an ideal electrolyte for MFCs. Its high content of inorganic salts translates into a high electrical conductivity value (160–270 mS/m), a property of electrolytes that considerably decreases ohmic losses and, consequently, reduces the internal resistance of MFCs, favoring power generation. On the other hand, the content of organic compounds or matter present in the HU expressed in COD represents the potential of chemical energy that can be converted into electrical energy (Table A3 [71]. The COD content in HU is 7-20 times higher than the COD content found in domestic wastewater (160 to 850 mg/L), another waste effluent widely used in BES.

Another critical HU characteristic is its carbonate-bicarbonate pH buffering capacity, caused by urea hydrolysis, which helps maintain a pH without significant variations [71].

Due to its abundance and physicochemical characteristics (Table A3), HU has recently gained considerable interest in WW treatment using MFCs, especially in the past 20 years.

#### 3.4.2. Microbial Fuel Cell Configuration Using Human Urine

In the early days of the second decade of the 21st century, the use of HU in MFCs as an energy source was reported [7]. From this study, the number of publications in this novel proposal began to grow significantly [9]. The proposal has leaped in a short time, going from prototypes at the laboratory level to prototypes on a pilot scale in real scenarios.

Several MFCs configurations have been tested to achieve the goals of each investigation. The HU characteristics used as a substrate and the types of inoculum utilized by researchers also represent important variables (Table 3).

Ieropoulos et al. [7] reported using HU in two types of procedures and settings. They used a bicameral MFC (Bi-MFC) with catholyte and anolyte recirculation and a single chamber MFC (SC-MFC) operated in batch mode. The current output reached and reported by the authors was 8 ± 0.5 mA/m^2^.

In the same year, Kuntke et al. [72] achieved ammonia recovery at a rate of 3.29 g N/day m^2^ from HU, and synthetic HU achieved a current density of 0.50 A/m^2^.

On the other hand, Zang et al. [81] designed an experiment to recover nutrients from HU. A SC-MFC with a proton exchange membrane was used in this experiment. Activated sludge was utilized as inoculum, whereas pretreated HU reduced in P and N, hydrolyzed and diluted HU added with magnesium sulfate, and disodium phosphate were the substrates. The power generated ranged between 0.1 and 0.325 mW. The reported removal efficiencies were 42.6 and 40%, respectively. Besides, a COD removal of 62.4% was obtained, a power density of 0.9 W/m^3^, and a percentage of 95% of struvite was found as a precipitate.

In 2013, the first SC-MFC without a membrane operated in batch mode with HU, as reported by Santoro et al. [8]. The authors utilized a modified glass bottle with a side hole where the cathode was connected. This component was tested with and without Pt coverage. The initial current generated by the SC-MFC of cathodes without Pt was 0.13–0.15 mA and stabilized at 0.1 mA. The cathode device with Pt decreased from 0.18–0.23 mA to 0.13 mA. This study showed that the high pH caused by the hydrolysis of urea reduced anodic reactions and the device’s overall performance. In a subsequent experiment using the same configuration, a power generation of 55 mW (without Pt) and 23 mW (with Pt) was achieved. Up to 75% of the COD in the urine was reduced after treatment. The ammonium concentration increased significantly, up to 5 g/L. The calcium and magnesium concentrations decreased due to the precipitation and high pH, and P decreased by 50% due to the formation of struvite on the cathode surface and at the bottom of the anodic chamber. The concentration of the ammonium ions increased four times due to the hydrolysis of urea. The bottom precipitation contained struvite, potassium, and hydroxyapatite.

Ieropoulos et al. [73] evaluated the serial connection of SC-MFCs in stacks of three devices, forming four cascades. This design used HU previously inoculated with acetate and yeast extract as a substrate. The output power of the stacks was in the range of 2–2.5 mW. The 12-cell pack could charge a basic mobile phone (MP).

One year later, Santoro et al. [82] reported a membraneless SC-MFC with low-cost activated carbon gas diffusion cathodes. The cells were fed HU, MWW, and WW with sodium acetate, phosphate buffer, and sodium acetate. The percentage of P eliminated was 40.1% in the case of urine feeding, which decreased to 15.7, 14.1, and 2.5% in the cases of WW + NaOAc, WW, and PBS + NaOAc, respectively. In terms of cathode performance and power output, the synthetic WW (PBS + AC) outperformed the raw sewage. However, no nutrients were removed or transformed. The output power of HU-powered devices was three times higher than raw WW and 25% higher than WW + NaOAc. Urine-fed MFCs reduced P content by 40%.

You et al. [83] used 15 interconnected MFCs. Two different materials for the anode (carbon veil and carbon cloth) were modified with a micropore layer. A commercial cation exchange membrane was sandwiched between the cathode and anode frames. The substrate consisted of HU inoculated with activated sludge. When the biofilm of the anodes matured, the maximum power output of the modified anodes was 304.3 mW (60.7 mW/m^2^) and 253.9 mW (50.6 mW/m^2^), using carbon veils and carbon cloth, respectively. It is the first case in which the micropore layer has been used efficiently for the anode.

On the other hand, Taghavi et al. [84] reported an operating power density (77 W/m^3^) in an experiment using three types of tubular SC-MFCs. This power density was reached after four weeks of operation. A year later, this group of researchers reported the first self-sufficient system powered by portable HU-operated MFC. The maximum potential reached was 110 μW, generated with a load of 30 kΩ. His design consisted of 12 pairs of MFCs (per leg) positioned in series, connected in parallel to the structure of a pair of socks. They, in turn, connect to an HU delivery pump located under the heels.

Pasternak et al. [85] highlight the importance of biofilm in producing bioelectricity. Additionally, ceramic and mullite membranes are good choices over commercial membranes. In their publication, cylindrical ceramic SC-MFCs of four different types interconnected in parallel were used. Energy production reached a density of 6.85 W/m^3^ with ceramic separators. HU enriched with electroactive bacteria from activated sludge was used as a substrate.

In the same year, Winfield et al. [86,87] published two investigations using paper and cylindrical and tetrahedral MFCs, interconnected in parallel. They used three types of inoculum for HU in their first publication: anolyte from an MFC operated with HU and fresh HU, primary effluent from WW and fresh HU, and finally, fresh HU. In this study, two paper MFCs, in parallel, transmitted radio signals for more than 24 h. These results proved that MFCs using HU could be useful to transmit “proof of life” in extreme situations. The paper separators reached a maximum power of 50–60 μW. In its second publication, the biodegradable MFCs stack could supply power to various devices for six months. A practical application for this technology includes onboard power supplies for biodegradable robotic systems. Biodegradable MFCs made with polylactic acid, natural rubber membranes, and lanolin-coated egg-based aerated cathodes were used in this experiment. Forty devices were operated in various configurations.

Shreeram et al. [88] documented the first observation of a urine-driven MFC operating with a genetically modified bacterial strain. A pilT mutation from the Gram-negative bacterium Pseudomonas aeruginosa showed a 2.7-fold increase in maximum power density compared to the wild-type strain, PAO1. It was determined that the high internal resistance observed near the open-circuit voltage is attributed to slow redox reactions at the anode surface and not to slow bacterial metabolism.

Another work to be highlighted is by You et al. [75]; in their publication, a system of SC-MFCs interconnected by gravity was reported. They used activated sludge mixed with yeast and tryptone as inoculum for its operation. This 3-stage system removed 82% of the TP and 20% of the COD from undiluted HU. In addition, 14.32 and 11.76 W/m^3^ of power were produced from the first and third stages of the system, respectively, during operation. In the same year, another investigation using interconnected SC-MFCs was reported. Small-scale cells were experimented with by varying the length of the electrodes. Their results reported doubling the electrode length, which multiplied the current density from 0.053 to 0.580 W/m^3^ [89].

Social applications orientation, initiated by Winfield in 2015 [86], by utilizing bioelectricity to transmit “proof of life”, were complemented by addressing environmental purposes. In 2017, investigations on MFCs by Ieropoulos et al. [90] reported the inactivation of pathogens (S. typhimurium, S. enteritidis, Salmonella typhimurium, and P. aeruginosa) using small-scale, single-chamber devices. They found that the bactericidal properties of the anode depended on the power output and the MFC redox potential. Besides, Pasternak et al. [91] utilized MFC bioelectricity to operate a self-powered biological oxygen demand (BOD) sensor for monitoring water quality. These applications evidence the tendency of the scientific community to address environmental issues from a sustainable perspective, addressing social, environmental, and economic issues.

Brewster et al. [92] modeled a bio-electroconcentration process, proposing a device with three chambers separated by cation and anion exchange membranes. His research objective was to recover N and P from synthetic ureolysed urine. The removal of TAN and total carbonate carbon (TCC) was between 43–57% and 39–53%, respectively. The current density reported was 90 A/m^2^.

Ledezma et al. [93] presented a novel bio-electroconcentration system (BEC), a hybrid MEC/ED cell. This reactor was designed to recover ammonia, phosphate, and K using synthetic urine. It consisted of three chambers separated by a cationic and an anionic membrane. The current density reached was 37.6 A/m^2^. These conditions favored the removal and recovery of the mentioned nutrients, besides the recovery of ammonium bicarbonate crystals with a content of 17% N.

Walter et al. [94] report the installation of the Pee Power^®^ device at the UK’s largest music festival. Its design consisted of 12 self-lit urinal modules powered by urine-operated MFCs.

On the other hand, Gajda et al. [79] reported an increase in catholyte production and pH in treating HU using an electrocatalyst derived from metal and carbon-containing iron and nicarbazine in cylindrical, single-chamber terracotta MFCs.

Santoro et al. [95] carried out the first study on MFCs using recycled paper as a supercapacitor. The maximum power presented was 1380 ± 0.083 mW (0.092 ± 0.006 mW/mL).

On the other hand, Salar-García et al. [96] reported using an iron-based catalyst (Fe-STR), reaching a maximum output power density of 104.5 ± 0.0 μW/cm^2^ in the cathodes with Fe-STR.

Sharma et al. [97] evaluated the MFC performance using a consortia mixture and pure cultures isolated from Firmicutes and Proteobacter species. This study focuses on microbial characterization, nutrient recovery, and electricity generation from pure and diluted HU. It was possible to verify that the microbes present in HU use less than 10% of the total phosphorus for their growth, while 90% is recovered as struvite. Microbial characterization showed that not all biofilms are efficient for bioelectricity production.

Finally, Walter et al. [98] reported using an MFC system that directly and continuously fed a microcomputer and its screen; this architecture produced an average of 62 mA and 158 mW.

The HU potential as a raw material from which it is possible to recover water, nutrients, and energy has been reflected in several recently published reviews focused on technologies operating with this anthropogenic waste to recover byproducts (Table 4). However, none of them addresses HU from the CE perspective.

**Figure 6:**
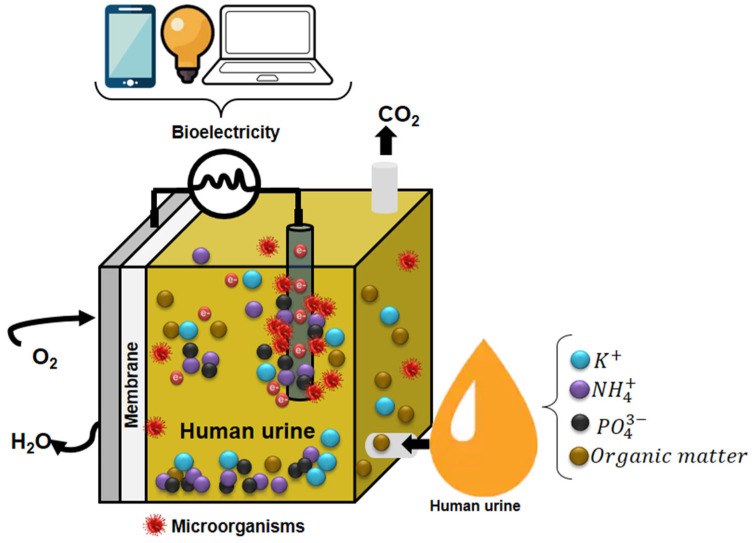
Devices powered by microbial fuel cells are fed with human urine.

According to Sadin et al. [106], the commercial suitability of a technology is determined by a classification system based on its level of technological readiness (TRL). Concerning this classification, MFCs are in the TRL1-TRL3 region, meaning their scientific development is ongoing at the laboratory level. In recent years, it has been widely demonstrated that MFCs fulfill useful functions without requiring external power to operate [107]. The MFCs have been trialed to demonstrate their ability to supply enough power to operate various devices (Figure 6, Table 5).

The potential shown by *MFC*s using *HU* as an alternative energy source has motivated pilot-scale trials in real-world scenarios.

#### 3.4.3. Real-Life Scenarios Trials

Driven by HU MFCs have been tested in real-life scenarios in an effort to try to elevate their TRL of MFCs.

The University of KwaZulu-Natal, Durban, South Africa, implemented a small-scale 66 MFCs configuration. The devices were batch fed for approximately 780 h with fresh HU. This design produced an average power density of 1.46 W/m^3^, which gradually decreased over time. Besides, the University of West of England (UWE) Frenchay campus in 2015 implemented an MFC system. The MFC configuration consisted of 288 MFCs. This configuration objective, commercially known as the Pee Power^®^ urinal, was to evaluate the technological parameters of the system and identify the limitations in a real-life scenario. The results showed that with five days of biofilm development, the MFC generated enough energy to light four domestic LED luminaires connected to a motion sensor powered by HU-operated MFCs. The power accumulates in supercapacitors, keeping it available. This trial implementation was executed in collaboration between UWE and the Oxford Committee for Famine Relief (Oxfam). The participation of the international confederation was motivated by the interest in applying the Pee Power^®^ urinal in refugee camps [107].

On the other hand, in 2015, a new study with the Pee Power^®^ urinal was developed using ca. 250,000 people attended a massive music festival. The configuration, similar to that implemented at the UWE, was complemented by four additional packages of MFCs connected to a more extensive urine flow system. The system contained 330 L of urine and fed six LED modules at night for 6 h. An average COD removal efficiency (η_COD_) of 30% was reported. One year later, another trial was carried out with changes to the device configuration, but with society witnessing real-time bioelectricity production. The modification consisted of reducing the urinal stands and installing a passive feeding mechanism that controlled the hydraulic retention time. Taking advantage of the laboratory experience reported by Walter et al. [109], membraneless, single-chamber MFCs operating in a self-straightening column were implemented. This system consisted of 12-cell modules, an energy harvesting and distribution system, and a passive power mechanism. This configuration powered six commercial LED tubular lamps with a modified voltage of 2650 V DC for 9.5 h/day when placed inside the urinal cubicles. This new configuration achieved a η_COD_ 37% higher than reported in the 2015 study [9]. The importance of these massive trials relies upon the fact that society is becoming aware that anthropogenic waste needs to be revalorized as a means of alleviating some of the most pressing global environmental problems and to mitigate adverse situations for the most vulnerable.

The reported works on MFCs operated with HU have focused on bioelectricity generation and nutrient recovery, opening the door to a new field of study focusing on water recovery.

## 4. Discussion

### Human Urine Oxidation by Electrolysis Systems

Unlike BES, specifically MFCs, an electrolysis device requires an external source of electrical current, i.e., its operation depends on external energy consumption. 

Electrochemical devices have several advantages in that they operate at ambient pressure and temperature. Furthermore, they show high performance and adaptability to variations in composition of waste effluents and flow rates. Another advantage of these devices is that they do not require auxiliary chemicals to operate and do not produce waste. The versatility of this technology allows it to be adapted to various applications and their coupling with other technologies. However, the cost of the electrodes and the generation of toxic byproducts in the treated water are some of its limitations [110].

Electrochemical treatments are a good option for decentralized wastewater treatment because they are controlled by electrode potential cell current. These operating control mechanisms are simpler to operate than conventional processes, i.e., chemical and biological processes. Furthermore, electrochemical treatment systems have versatile advantages, such as their compatibility with most of the existing technology for wastewater treatment and their adaptability to variations in the composition of the wastewater to be treated [110].

Some processes, such as RO, NF, MD, and FO, have been used to increase the concentration of nutrients in HU (Table A2). On the other hand, for the recovery of struvite ion exchange, electrodialysis, and BES have been applied [25]. These advantages are concomitant with water CE.

Electrolysis has proven to be a viable alternative to polishing waste effluents when they present slight coloration and content of organic material (around 30–40 mg COD/L, making them more acceptable and increasing their level of safety [111]. Another application of electrochemical treatments is the removal of residual chlorine and disinfection byproducts as a terminal treatment for recovered water when the objective is direct consumption [110]. On the other hand, the residual effluent produced by electrochemical systems must be treated to restore its properties (i.e., pH, redox potential) before being discharged into the biosphere [110]. One of these processes is chlorine-producing electrolysis [111]. To optimize energy consumption (ca. 30 Wh/day) in these processes, solar panels connected to batteries have been used in the back of the toilets [111].

Electrolysis has been a method used to remove nitrogen from urine [112]. In recent years, the electrochemical oxidation of ammonia and urea has gained attention [113]. On the other hand, electrolysis has also functioned as an ammonia sensor in effluents and for the recovery of hydrogen and urea [114,115]. In the treatment of MWW, the most common mechanism for electrochemical oxidation of ammonia is indirect oxidation with chlorine [116]. The above means that it is possible to harvest hydrogen through electrolysis, which is considered the future fuel because it does not generate CO_2_ as a product of its combustion [117].

The type of treatment required depends on the specific nutrients to extract, e.g., the decrease in pH by an electrochemical method in combination with a magnesium-titanium corrosion cell allows the separation of nitrogen and phosphorus by variation of voltage.

One limitation when using HU separated from the source is the unpleasant odor it gives off and the corrosion from ammonia release, which could be a limitation for social acceptance of this technology. Ikematsu et al. [118] explored the electrooxidation process at a Pt/Ir anode, achieving odor reduction.

#### Electrodialysis

Electrodialysis (ED) has been utilized to treat different residual effluents, i.e., the removal of salts, the recovery of organic acids, and the treatment of DW, among other applications [119] (Table 6).

In treating HU by ED, the membrane functions as a physical barrier to retain bacteria, viruses, and pathogenic proteins [119]. Results obtained by Goodman et al. [121] are clear evidence that hybrid technologies focused on water CE can lead to water quality restoration; in this research, reclaimed water was intended for irrigation purposes (Table 6). Furthermore, Ghernaout and Elboughdiri [126] state the necessity to scale up WW treatment to achieve the highest purity level, i.e., potabilization, by adding treating processes such as nanofiltration, reverse osmosis, and adsorption on activated carbon. Additional treatment would lead to additional costs; therefore, the social consciousness of environmental detriment, particularly in water shortages, is a must. By achieving social acceptance of reclaimed water, it would be easier to allow nature to regenerate by stopping the extraction of virgin resources, as claimed by water CE.

Patel et al. [25], support the idea of integrating at least two technologies to achieve water recovery and concentrate nutrients in HU efficiently; FO is pointed out as the best technology to achieve water reclamation from treated HU.

The reported works on MFC have primarily focused on bioelectricity generation and nutrient recovery, opening up a field for water recovery research.

Despite the different revaluation approaches reported by the scientific community for HU, its use remains a subject associated with prejudice. However, today, HU is challenging science and society to become unconventional resources. The popularity of this residue attributes increasing recognition to it, associating it with great economic and environmental benefits [31].

## 5. Outlook

The world demand for freshwater, an essential resource for life but limited by its abundance and quality on the planet, has increased directly due to the demographic explosion. This has triggered the scientific community to seek unconventional resources to renew, recover, or reuse water to increase its efficiency of use. These actions are within the water CE philosophy.

Residual effluents are a problem from the current perspective. This vision can be modified depending on WW composition and the possibility of recovering byproducts with socio-environmental interest. For society, the HU is an unconventional resource due to its advantageous composition. It is considered one of the principal sources of macronutrients for plants. Furthermore, its organic matter content can be converted into energy using BES. Additionally, it represents an unconventional water source through WWTP or MFCs, directly or indirectly, from the transpiration of plants.

From the water CE concept, WW or HU could be the raw material for other processes to obtain products such as hydrogen, methane, fertilizers, electricity, and water. MFCs are an interesting technology that can contribute to the reintegration of HU as a source of nutrients, energy, and water within the productive sector, i.e., through this technology, the valorization of HU will change. It will no longer be a waste without value. It will become a product of social interest.

Even though MFCs operated with urine as an anolyte do not generate attractive potential outputs or produce considerable amounts of bioelectricity, the results are promising. Electro and bioelectrochemical systems allow urine to be pretreated for secondary processes that can be coupled to recover water. 

Even when MFCs are in the RL1- TRL3 region, scientific evidence shows the potential of this technology to revalue HU when coupled with conventional WW treatment technologies. In recent years, it has been widely demonstrated that MFCs perform valuable functions without requiring external energy to operate when using HU as fuel.

Nutrient recovery as N, P, and K is easier from source-separated HU than from WW. Retrofitting hydrosanitary facilities to separate HU from WW contributes to urine recovery for different treatments to obtain byproducts such as water, bioelectricity, and nutrients like N, P, and K from a less complex matrix more efficiently. Today, unconventional water, nutrients, and energy sources are needed; HU is one of them.

## Figures and Tables

**Table 1:** Treatment methods for human urine.

Treatment	Pre-Treatment	Operational Conditions	Results	Reference
RO ^a^	Sieving through a 0.5 mm sieve, filtering with a 5 mm cartridge filter, and UF ^b^ with a cut-off of 100,000 Daltons	20 to 45 °C pH 4.5 to 7 Electrical energy required = 8 kWh/L	1 m^3^ urine produced 200 L of concentrate at pH 7 with 95% N, 90% P, and 95% K	[50]
DC-MD ^c^	Filtration of acidified urine by membrane filter paper of 1.2 mm pore-size	Feed temperatures of 40, 50, 60, and 70 °C Fixed permeate temperature = 20 °C pH 5, 6, 7, 8, and 9 TAN ^d^ concentrations: 0.465, 0.986, 1.945, 2.972, 3.972, and 4.940 g-N/L	TAN was successfully concentrated with a low specific ammonia transfer value (<2.06 × 10^−3^ g-N/g-H_2_O)	[51]
	Urine hydrolyzation	pH 10.5, water vapor gradient of 30 °C. Feed temperature = 50 °C and the permeate temperature = 20 °C	Water recovery of 80% The highest permeate flux for the PVDF/PTFE ^e^ nanocomposite membranes was 8 J/Jo The permeate water quality was: rejection of ammonia (>95%), total organic carbon (TOC) ^f^ (>98%), Na^+^ (>98%), and K^+^ (>89%)	[40]
FO ^g^	Filtered through a 0.2 micron filter for sterility 1250 U of urease were added stirring overnight at room temperature	Hydrolyzed and unhydrolyzed artificial urine feed solutions using NaCl or glucose DA ^h^	Urea hydrolysis, in combination with lowering the feed solution pH, significantly improved TN ^i^ rejection. The rejection efficiency of $NH_{4}^{+}$ was almost 100%	[52]
Hybrid (FO-MD ^j^)	Undiluted HU ^k^	The initial volumes of the feed and draw (NaCl) solutions were 0.6 and 0.5 L, respectively. The operating time was 2 h. The inlet temperatures of the urine feed were set at 45 and 53 ℃	$\mathrm{Leakage}\;\mathrm{rates}\;\mathrm{of}\;\mathrm{TOC}{\;}^{f},\;\mathrm{TN}{\;}^{i},\;\mathrm{and}\;NH_{4}^{+}$-N are no more than 0.7, 1.1, and 0.2%, respectively in 2 h. $\mathrm{Rejection}\;\mathrm{of}\;NH_{4}^{+}$-N above 99.5%	[53]
Evaporation	NA ^l^	Low-cost heat source using solar thermal techniques	360 g solid fertilizer from 50 L undiluted HU within 26 days	[54]
Lyophilization	NA	FTM ^m^ together with struvite recovery and nitrogen adsorption on zeolite, and AC ^n^	The FTM method concentrated 60% of the nutrients in 40% of the initial volume and significantly improved the N reduction. The P recovery was 95–100%, mainly as struvite.	[55]
		Struvite crystallization with ammonia adsorption on zeolite and wollastonite	Clinoptilolite, wollastonite, and a natural zeolite showed excellent adsorbent properties in contact with ammoniacal solutions. In combination with struvite crystallization, 65 ± 8% of the N was recovered as crystalline or adsorbed ammonium. Concentrated the nutrients from urine up to 80% of the original volume at a temperature of −14 °C	[56]

^a^ Reverse osmosis; ^b^ Ultra-filtration; ^c^ Direct contact membrane distillation; ^d^ Total ammoniacal nitrogen; ^e^ Polyvinylidene fluoride/polytetrafluoroethylene; ^f^ Total organic carbon; ^g^ Forward osmosis; ^h^ Draw agents; ^i^ Total nitrogen; ^j^ Forward osmosis-membrane distillation; ^k^ Human urine; ^l^ No apply; ^m^ Freezing-thawing method; ^n^ Active carbon.

**Table 2:** Available human urine treatment techniques advantages and disadvantages.

Human Urine Treatment Techniques	RO ^a^	NF ^b^	MD ^c^	FO ^d^	Ev ^e^	Ly ^f^
**Advantages**						
Effectively concentrates nutrients	X	X	X	X	X	X
High rejection of micropollutants	X	X		X		
Rapid concentration time and separation of water	X	X		X		
Separates good quality water			X			
High rejection of pathogens				X	X	
Significantly less energy is required (recirculation/pumping)				X		
**Disadvantages**						
Consumption of a significant amount of energy	X	X	X		X	X
Poor feasibility for long-term operation	X	X				
Presence of salts, heavy metals, and micropollutants	X	X				
Scaling of the membrane is the prime issue	X	X		X		
Loss of N and/or valuable elements			X		X	
Volatile products can contaminate the permeate			X			
Regeneration of the DA ^g^ is challenging				X		

^a^ Reverse Osmosis; ^b^ Nanofiltration; ^c^ Membrane distillation; ^d^ Forward osmosis; ^e^ Evaporation; ^f^ Lyophilization; ^g^ Draw agents.

**Table 3:** Microbial fuel cells operating with actual human urine.

MFC ^a^ Configuration	Inoculum	I_MFC_^b^	η_Coul_^c^ (%)	P_MFC_^d^	Recovery/ Removal		η_COD_^g^ (%)	Reference
					TN ^e^	TP ^f^		
Two-chamber; membrane electrode assembly	Effluent of another active MFC operated on synthetic media with acetate	0.50 A/m^2^	10	2500 µW	3.29 g N/d m^2^	NR ^h^	NR	[72]
Bicameral, recirculating MFCs with cation exchange membrane operated in batch mode	Microflora from activated sludge	0.25 mA	NR	108 µW	NR	NR	NR	[7]
Membrane-less; ceramic separator; plain carbon-based electrodes	Anaerobic sludge, supplemented with acetate and yeast-extract	2900 µA	NR	2180 µW	NR	NR	NR	[73]
Stacks of small-scale MFCs	NR	NR	NR	1500 µW 5500 µW/m^2^	NR	NR	NR	[74]
Membraneless single-chamber MFCs; Pt-based or Pt-free cathode s	Raw WW ^I^ and sodium acetate solution	0.18–0.23 mA	NR	23 µW	NR	20–50%	60–75	[8]
Two MFC groups, each group had four MFCs units; cation exchange membrane	ASS ^j^, yeast extract, and tryptone	NR	NR	358 μW 14.32 W/m^3^	20%	82%	20	[75]
Single-chamber clay MFCs; four non-fluorinated polymers tested as binders to PTFE ^k^	ASS and HU ^l^	2250 µA	NR	510 µW	NR	NR	26	[76]
Bicameral MFC; batch operated; acclimation method	Filtered anaerobic sludge from WWTP ^m^	495 mA/m^2^	26.5%	NR	NR	NR	75.5	[77]
Stack, mullite cylindrical single chamber MFCs	50% ASS and 50% HU	NR	NR	800 μW	NR	NR	NR	[78]
560 Cylindrical terracotta MFCs; modular stack configuration	1:1 activated sludge and urine mix	70,000 µA	NR	20.4 W/m^3^ 245 mW	NR	NR	NR	[79]
Small-scale ceramic MFC; metal–carbon-derived electrocatalyst	1:1 activated sludge and urine mix	NR	NR	1990 μW 44.8 W/m^3^	NR	NR	>75	[80]
Dual chamber plate and frame type MFC	Heat-treated and diluted digestate from an anaerobic digester	0.461 A/m^2^	NR	0.68 W/m^2^	54.28%	94.2	69.97	[13]

^a^ Microbial fuel cell; ^b^ Microbial fuel cell current intensity; ^c^ Coulombic efficiency; ^d^ Microbial fuel cell power output; ^e^ Total nitrogen; ^f^ Total phosphorus; ^g^ Chemical oxygen demand removal efficiency; ^h^ Not reported; ^i^ Wastewater; ^j^ Activated sewage sludge; ^k^ Polytetrafluoroethylene; ^l^ Human urine; ^m^ Wastewater treatment plant.

**Table 4:** Recently published reviews related to human urine treatment for byproduct recovery.

Title	Reference
Recent progress on the recovery of valuable resources from source-separated urine on-site using electrochemical technologies: A review	[99]
Bioelectricity generation from human urine and simultaneous nutrient recovery: Role of Microbial Fuel Cells	[100]
Membrane technologies in toilet urine treatment for toilet urine resource utilization: a review	[101]
State of the art of urine treatment technologies: A critical review	[102]
Urine in Bioelectrochemical Systems: An Overall Review	[9]
Urine treatment technologies and the importance of pH	[103]
Technologies for the recovery of nutrients, water, and energy from human urine: A review.	[25]
Waste or Gold? Bioelectrochemical Resource Recovery in Source-Separated Urine	[104]
Moving towards practical applications of microbial fuel cells for sanitation and resource recovery	[105]

**Table 5:** Feeding on urine microbial fuel cells practical applications at laboratory scale.

Reactor	Device Fed	Results	Reference
24 ceramic membrane-less MFCs ^a^ stack; carbon-based electrodes	Samsung GT-E2121B MP ^b^	- Mobile phone charged to 3.7 V: >4 min outgoing call - 2 mW	[73]
6 abiotic; origami tetrahedron MFCs stack	Portable ELTs ^c^	- 238 radio signals broadcasted over 24 h	[86]
Fully biodegradable 40 MFC stack	Red LED ^d^ (HLMP-D150, Avago Technologies)	- 246 µW or 6.16 W/m^3^	[87]
Wearable MFC; urine Foot-pump	Self-sufficient wireless transmitter	- 110 μW, with a connected load of 30 kΩ - Circuit output voltage:4 V	[84]
24 MFCs stacks; 8 MFCs of 6.25 mL anodic volume each	Commercially available electronic air freshener	- 82 P and 20% COD ^e^ removal - 14.32 W/m^3^ - 358 μW	[75]
Gravity fed MFC cascade; 6 modules with 20 MFCs each	- Smartphone - Remote system charging	Mobile phone charger based on urine: 3 h outgoing call; 6 h of charge/600 mL	[108]
4 terracotta cylinders SC-MFCs ^f^; connected in parallel	BOD ^g^ biosensor for online water quality monitoring	- Urine detection in fresh water - Visual and sound cues (85 dB). - 25.4 μW.	[91]
Hybrid 3-chamber reactor: ED ^h^ system and MFC	Self-powered bioelectrochemical nutrient recovery system	- 3 A/m^2^ for >2 months - simultaneous upconcentrating N and K by a factor of 1.5–1.7.	[28]
4 self-stratifying membrane-less MFCs modules cascades	Microcomputer and its screen (Gameboy Color, Nintendo^®^)	- 62 mA at 2550 mV - 158 mW)	[98]

^a^ Microbial fuel cell; ^b^ Mobile phone; ^c^ Emergency locator transmitters; ^d^ Light-emitting diode; ^e^ Chemical oxygen demand; ^f^ Single chamber microbial fuel cells; ^g^ Biological oxygen demand; ^h^ Electrodialysis.

**Table 6:** Electrodialysis treatment.

Process	Purpose	Effluent	Results	Reference
ED ^a^	Salts and ion removal	Diluted HU ^b^	Removal of ion was nearly complete without amino acid loss	[120]
Membrane desalination system based on EDR ^c^	Salts and TDS ^e^ removal	Treated MWW ^d^	The EDR ^c^ process reduced the TDS ^e^ from 1104 to 328 mg/L. Reductions: conductivity 72%, calcium 84%, chloride 76%, fluoride 59%, alkalinity 64% and phosphate 60%; Power consumption = 0.6 kWh/kL; and media filtration 0.4 = kW/kL. Total operating cost = 18 cents/kL to deliver 82% water recovery	[121]
ED ^a^ stack	Nutrient concentration and micropollutant retention	Synthetic and pretreated natural HU ^b^	Ethinylestradiol removal. Propranolol and ibuprofen were initially removed but broke through during EOT ^f^. Diclofenac and carbamazepine retention = 90–95% at EOT ^f^	[119]
Two-compartment EDBM ^g^	Organic acid recovery (Lactic acid)	Real fermentation broth	Retained ion efficiency = 98.6% Lactic acid concentration = 29.7–156.8 g/L (c = 0.33–1.74 mol/L), corresponding to 85–98% of conversion, was obtained; the energy consumption was ca.1.1 kWh/kg	[122]
Two-compartmen ED ^a^ unit (10 cells) with membrane stack		Sodium lactate	Lactate recovery ca. 100% in a batch recirculation operation mode for current density values < 400 A/m^2^. Current efficiency > 95%	[123]
EDR ^h^	DW ^i^ treatment, Ions, and EP ^j^ removal	Raw water from the Mediterranean Llobregat River	Ionized compounds, partially removed. EP ^j^ removals >65% and often beyond 90% for the overall DW treatment plant process.	[124]
ED ^a^ with ion-conducting intermembrane spacer	DW treatment Nitrates reduction	Synthetic DW	Selective removal of nitrates from DW. 65% elimination of the total nitrate content. The nitrate concentration is reduced from 93 to 32 mg/L	[125]

^a^ Electrodialysis; ^b^ Human urine; ^c^ Electrodialysis reversal; ^d^ Municipal wastewater; ^e^ Total dissolved solids; ^f^ Extended operating times; ^g^ Electrodialysis with bipolar membranes; ^h^ Electrodialysis reversal; ^i^ Drinking water; ^j^ Emerging pollutants.

## Data Availability

Not applicable.

## Appendix A

**Table A1:** Water volume occupied for the discharge of excreta according to the type of toilet used.

Toilet Type	Water Consumption (L/Discharge)			References
	General Discharge	Large Discharge	Small Discharge	
Conventional toilet flush systems	6–12	*NA* ^a^	*NA*	[45,127]
Dual flush systems	*NA*	4–6	2–3	
Vacuum-flushed toilets	0.5–2	*NA*	*NA*	
Very low flush with gravity sewers	0.6–1	2	0.2	
Urine-diverting flush toilet	*NA*	4–6	0.2	[45]
Urine-diverting dry toilet	*NA*	*NA*	*NA*	[41]

^a^ No apply.

## Appendix B

**Table A2:** Recovery techniques of nutrients from urine.

Recovery Technique	References
Lyophilization	[56,68,128]
Volume reduction by reverse osmosis	[51,129]
Nanofiltration	[57,130]
Forward osmosis	[61,62]
Membrane distillation	[51,128,131]
Stabilization and distillation	[132,133,134]
Ion exchange	[71,135,136,137]
Electrodialysis	[119,138]
Volume reduction by evaporation	[54,66,139]
Struvite formation	[131,140,141,142,143,144,145]
Air stripping for ammonia recovery	[53,72,146,147,148,149]
Microbial electro-concentration	[19,93]

## Appendix C

**Table A3:** Principal solutes in human urine.

Solutes	Concentration (g/L)
Urea	9.30–23.30
Inorganic salts	14.16
Organic ammonium salts	4.13
Organic compounds	5.36
Chemical oxygen demand	6.30–17.50
Total nitrogen	4.00–13.90
Total phosphorus	0.20–2500
Potassium	0.45–1.44
